# Psychiatric Comorbidity, Social Aspects and Quality of Life in a Population-Based Cohort of Expecting Fathers with Epilepsy

**DOI:** 10.1371/journal.pone.0144159

**Published:** 2015-12-04

**Authors:** Simone Frizell Reiter, Gyri Veiby, Marte Helene Bjørk, Bernt A. Engelsen, Anne-Kjersti Daltveit, Nils Erik Gilhus

**Affiliations:** 1 Department of Clinical Medicine, University of Bergen, Bergen, Norway; 2 Department of Neurology, Haukeland University Hospital, Bergen, Norway; 3 Department of Global Public Health and Primary Care, University of Bergen, Bergen, Norway; 4 Medical Birth Registry of Norway, Division of Epidemiology, Norwegian Institute of Public Health, Bergen, Norway; Catholic University of Sacred Heart of Rome, ITALY

## Abstract

**Objectives:**

To investigate psychiatric disorders, adverse social aspects and quality of life in men with epilepsy during partner’s pregnancy.

**Method:**

We used data from the Norwegian Mother and Child Cohort Study, including 76,335 men with pregnant partners. Men with epilepsy were compared to men without epilepsy, and to men with non-neurological chronic diseases.

**Results:**

Expecting fathers in 658 pregnancies (mean age 31.8 years) reported a history of epilepsy, 36.9% using antiepileptic drugs (AEDs) at the onset of pregnancy. Symptoms of anxiety or depression were increased in epilepsy (7.0% and 3.9%, respectively) vs. non-epilepsy (4.6% and 2.5%, respectively, p = 0.004 and 0.023), and so were new onset symptoms of depression (2.0% vs. 1.0%, p < 0.031) and anxiety (4.3% vs. 2.3%, p = 0.023). Low self-esteem (2.5%) and low satisfaction with life (1.7%) were more frequent among fathers with epilepsy compared to fathers without epilepsy (1.3% and 0.7%, respectively, p = 0.01 and 0.010). Adverse social aspects and life events were associated with epilepsy vs. both reference groups. Self-reported diagnoses of ADHD (2.2%) and bipolar disorder (1.8%) were more common in epilepsy vs. non-epilepsy (0.4% and 0.3%, respectively, p = 0.002 and 0.003) and non-neurological chronic disorders (0.5% and 0.5%, respectively, p = 0.004 and 0.018). A screening tool for ADHD symptoms revealed a higher rate compared to self-reported ADHD (9.5% vs. 2.2%, p < 0.001).

**Conclusion:**

Expecting fathers with epilepsy are at high risk of depression and anxiety, adverse socioeconomic aspects, low self-esteem, and low satisfaction with life. Focus on mental health in fathers with epilepsy during and after pregnancy is important. The use of screening tools can be particularly useful to identify those at risk.

## Introduction

Pregnancy and birth generally have a positive impact on the parent’s life and well-being, but may also be associated with increased stress, anxiety, and other forms of psychiatric dysfunction. Early recognition of emotional distress in women during pregnancy and the post-partum period is important in order to prevent complications such as birth-anxiety and post-partum depression [1]. Expecting fathers are increasingly involved during and after pregnancy and studies suggest that they too may be predisposed to psychiatric dysfunction, including anxiety and depression during pregnancy and post-partum, as well as a general decline in mental health during the first year after birth [2–4]. Moreover, studies show that lack of support from the partner is a risk factor for emotional dysfunction in both parents [3] and this effect is stronger in the presence of pre-existing somatic or mental illness [5, 6].

Both men and women with epilepsy have an increased risk of psychiatric comorbidity, including depression and anxiety [7, 8]. Epilepsy has also been linked to ignorance and superstition, causing fear and stigma, with a negative impact on aspects such as education, employment, intimate relationships, and quality of life [9, 10]. This burden can add to a vulnerable situation for both fathers and mothers with epilepsy during pregnancy. Women with epilepsy face extra challenges related to antiepileptic drug (AED) -treatment in order to maintain seizure control during and after gestation [11]. They are also at higher risk of peri-partum depression or anxiety than women without epilepsy [12]. Although men are not physiologically influenced by childbearing, mental health during and after pregnancy can be affected by psychological factors such as insecurity about seizures and the new challenges of fatherhood. Our hypothesis is that the increased risk of psychosocial challenges in persons with epilepsy may add to a vulnerable situation during pregnancy for expecting fathers. This can be examined by our unique dataset, from which we have conducted a cross-sectional study based on a large national cohort of men with detailed self-reported data during their partner’s pregnancy.

## Material and Methods

### Data collection and assessment of diagnoses

This cross-sectional study included data from version 5 of the quality-assured data files based on The Norwegian Mother and Child Cohort Study (MoBa), conducted by the Norwegian Institute of Public Health[13]. A detailed description of the cohort has been published previously [13]. From the year 2000 the mothers’ partners were invited to participate, and 87% of the expecting fathers agreed to participate. The present study included all 76,335 pregnancies with data from the expecting fathers during gestational weeks 13–17, with detailed information on past and current psychiatric diseases, socioeconomic conditions, and AED use during the last six months prior to pregnancy.

The main control group to be included was expecting fathers without epilepsy. An additional group with non-neurological chronic disorders was chosen to assess whether associations found for epilepsy were epilepsy-specific rather than caused by the burden of a chronic disorder in general. The stratification into AED treated and untreated epilepsy is relevant as a marker for epilepsy severity and potential AED effects. AEDs could modulate psychiatric symptoms.

Fathers with epilepsy (FWE) comprised 658 unique pregnancies, and were classified according to AED use during the last 6 months (yes/no), and further divided into four main AED groups: Monotherapies with valproate (VPA, n = 59), carbamazepine (CBZ, n = 91), or lamotrigine (LTG, n = 40), and multiple AEDs (polytherapy, n = 30). The total epilepsy group were compared to a reference group of all fathers in MoBa without epilepsy (n = 75,677). A subgroup of 8,475 of the references had a non-neurological chronic disorder (NNCD), including diabetes, rheumatic arthritis, heart disease or asthma. This group served as a second reference group.

### Variables

The screening instruments were constructed as dichotomous variables. The instruments and measures are presented in S1 Table. Current depressive and anxiety symptoms were measured separately by a short version of the Hopkins’ Symptom Check List [14] with 4-items scales for depression (SCL-D) and anxiety (SCL-A). A mean score > 1.75 was set as cut off for significant depression or anxiety [14]. Previous depression was assessed by the Life Time Major Depression Scale (LTMD, S1 Table), a validated tool [15] which meets DSM III-criteria for lifetime major depression when i) at least three types of symptom items are endorsed, ii) one of these symptoms is the first (felt depressed), iii) three types of symptoms occurred simultaneously. Screening-positive ADHD symptoms were assessed by a 6-item short version of the Adult ADHD Self Report Scale (ASRS). ASRS has shown good internal consistency for use in both epidemiological and clinical surveys [16] (S1 Table). Quality of life was evaluated through the 4-item short version of Rosenberg’s Self Esteem Scale (RSES) and 5-item Satisfaction With Life Scale (SWLS) (S1 Table). The short version of RSES has shown a 0.95 correlation with the original 10 items scale [17, 18], and SWLS has also been validated as robust [19]. Low satisfaction with life was defined as SWLS score ≤ 9. Cronbach’s alpha was 0.81 for LTMD, 0.69 for SCL-D, 0.78 for SCL-A, 0.50 for ASRS, 0.71 for RSES and 0.86 for SWLS. A maximum likelihood estimation procedure for missing values was applied for the screening tools to avoid potential sample distortions [20]. Screening outcomes with ≥ 20% missing data were excluded from the analyses. Predefined psychiatric diagnoses in the questionnaire included: Attention Deficit Hyperactivity Disorder (ADHD) (yes/no), bipolar disorder (yes/no) anorexia/bulimia/other eating disorder (ED) (yes/no), and schizophrenia (yes/no). In addition the questionnaire included a box for unspecified (other) psychiatric disorders (yes/no). Paternal demographic and socioeconomic variables included age (years), low educational level (≤ 12 years), low income (≤ 26,704 Euro/year), unemployment due to disability (yes/no), current smoking (yes/no), high alcohol use during partner’s pregnancy (> ten units/week), and a history of narcotic use (yes/no). Narcotics included cannabis, amphetamine, ecstasy, cocaine, and heroin. Amphetamine recorded as a narcotic was assessed through specific questions on drug abuse separate from questions on use of medication in relation to ADHD. Low income was defined according to The European Commission, including a household income per consumption unit < 60 percent of the median [21, 22]. Financial insecurity was defined as not being able to handle unexpected expenses of 1,180 Euro in a month. 11 adverse life events were assessed by the question “Have you experienced any of the following (events) during the past 12 months?” (S1 Table) (yes/no).

During the MoBa inclusion period the questionnaires were modified several times, resulting in some of the variables being available only from later questionnaire versions (D and E), and numbers are lower for these analysis.

### Statistics

SPSS Statistics 21.0 (SPSS Inc., Chicago, IL, USA) was used to perform the analyses. Mean age was compared through independent-samples t-test. All other measures were constructed as dichotomous variables and were analyzed by Pearson’s chi-square test, and by Fisher’s exact test for cross-tabulations with expected cell count less than five. Results are presented as crude frequencies and unadjusted odds ratios (OR) with 95% confidence interval (CI) and corresponding p-values. Two-sided p-values < 0.05 were considered statistically significant. When significant differences were found between the epilepsy group and control groups through chi square testing, the differences were further tested with binary logistic regression analysis for potential classical confounders as well as socioeconomic conditions associated with epilepsy in our analysis. Results are presented with adjusted OR with 95% CI and corresponding p-values. OR, CI and p-values in all tables and figures refer to comparisons between the fathers with epilepsy and the main reference group without epilepsy. Significant differences between the epilepsy and NNCD groups are marked with “#” in the tables and figures. Age, low income, and low educational level were considered potential confounders. McNemar test was used to compare differences between dichotomous variables for diagnoses vs. symptoms.

### Ethics

The MoBa study and the current sub study have been approved by The Regional Committee for Medical Research Ethics in Western Norway (2010/788).

## Results

0.9% (n = 658) of the expecting fathers reported a history of epilepsy (Table 1), 36.9% (n = 243) having used AEDs during the last six months prior to partners pregnancy, the majority as AED monotherapy (87.2%, n = 212).

**Table 1:** Percentage and number (n) of individuals with various social characteristics in fathers with epilepsy with and without use of antiepileptic drugs (AEDs) compared to a reference group without epilepsy. Fathers with non-neurological chronic disorders (NNCD) served as an additional internal control group.

	References (n = 75677)	NNCD (n = 8475, 11.1%)	Epilepsy all (n = 658, 0.9%)			Epilepsy with AED (n = 243, 0.3%)			Epilepsy without AED (n = 415, 0.5%)		
**Characteristics**	**% (n)**	**% (n)**	**% (n)**	**p-Value**	**OR (CI)**	**% (n)**	**p-Value**	**OR (CI)**	**% (n)**	**p-Value**	**OR (CI)**
Mean age in years (SD)	32,3 (5.4)	32.1 (5.3)^##^	31.8 (5.6)	0.009	NA	32.1 (5.4)	0.14	NA	31.7 (5.7)	0.031	NA
Low education (< 12 years)	46.7 (35357)	49.3 (4144)	48.9 (322)	0.26	**1.1** (0.9–1.3)	48.1 (117)	0.66	**1.1** (0.8–1.4)	49.4 (205)	0.28	**1.1** (0.9–1.4)
Low income^1^	5.4 (1776)	5.9 (229)^##^	9.9 (27)	0.001	**1.9** (1.3–2.9)	10.3 (10)	0.031	**2.0** (1.1–3.9)	9.7 (17)	0.011	**1.9** (1.1–3.1)
Lack of financial security^1^	18.4 (6069)	20.8 (801)	22.5 (61)	0.079	**1.3** (1.0–1.7)	21.9 (21)	0.37	**1.2** (0.8–2.0)	22.9 (40)	0.13	**1.3** (0.9–1.9)
Unemployed due to disability	1.4 (1043)	2.8 (239)^##^	5.2 (34)	<0.001	**3.9** (2.7–5.5)	9.1 (22)	<0.001	**7.1** (4.5–11.0)	2.9 (12)	0.009	**2.1** (1.2–3.7)
Sick leave > 8 weeks^1^	6.2 (1244)	8.7 (215)	11.2 (21)	0.005	**1.9** (1.2–3.0)	15.3 (11)	0.005	**2.7** (1.4–5.2)	8.7 (10)	0.27	**1.4** (0.75–2.8)
Smoking	23.6 (17858)	24.6 (2065)	24.3 (160)	0.67	**1.0** (0.9–1.2)	19.3 (47)	0.12	**0.8** (0.6–1.1)	27.2 (113)	0.082	**1.2** (1.0–1.5)
Alcohol^1^	4.8 (1626)	5.4 (213)	5.4 (15)	0.65	**1.1** (0.7–1.9)	5.1 (5)	0.81	**1.0** (0.4–2.6)	5.6 (10)	0.63	**1.2** (0.6–2.2)
Narcotics ever	17.2 (13033)	19.4 (1634)	17.6 (116)	0.78	**1.0** (0.8–1.3)	12.3 (30)	0.044	**0.7** (0.5–1.0)	20.7 (86)	0.060	**1.3** (1.0–1.6)

Significant difference between the NNCD versus ‘Epilepsy all’ groups:

^##^p < 0.01.

Unadjusted OR, odds ratio; CI, confidence interval; SD, standard deviation; NA, not applicable.

^1^. Data from questionnaire version D and E only.

### Psychiatric symptoms and self-reported disease

**Fig 1:**
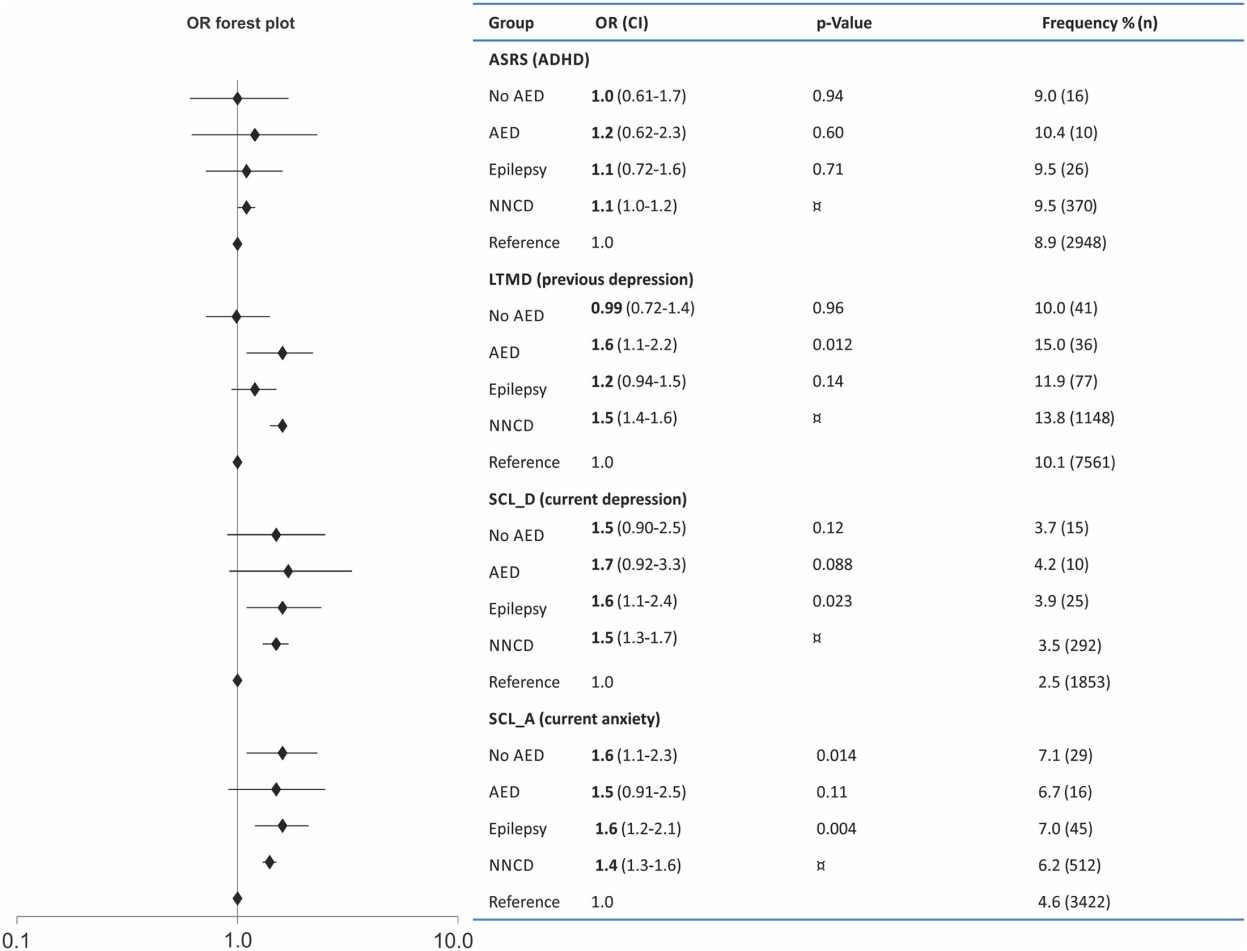
Frequencies for symptoms of ADHD tested with ASRS, previous depression tested with LTMD, current depression tested with SCL_D and current anxiety tested with SCL_A in fathers with epilepsy with and without use of antiepileptic drugs (AEDs) compared to a reference group without epilepsy. Unadjusted p-values and odds ratios (OR) are given for these comparisons. Fathers with non-neurological chronic disorders (NNCD) served as an additional internal control group. ¤ No significant difference between the NNCD versus ‘Epilepsy all’ groups. CI, confidence interval; SD, standard deviation; ASRS, Adult ADHD Self Report Scale; LTMD, Lifetime Major Depression Scale; SCL_D, Hopkins Symptom Check List for current depressive symptoms; SCL_A, Hopkins Symptom Check List for current anxiety symptoms.

During pregnancy, FWE more frequently reported symptoms consistent with anxiety (SCL-A) and depression (SCL-D) compared to the reference group, but not more frequently than fathers with NNCD (Fig 1). The association between epilepsy and anxiety was consistent after adjustment for confounders (OR = 1.7, p = 0.018), but not for depression (OR = 1.6, p = 0.13) (complete list of adjusted numbers for symptoms in S2 Table). Significantly more FWE had new onset symptoms of depression (2.0% vs. 1.0%, OR = 1.8, CI = 1.0–3.2, p < 0.031) and anxiety (4.3% vs. 2.3%, OR = 1.8, CI = 1.3 p = 0.023) during pregnancy compared to the references without epilepsy. Previous depression was more common in the AED-treated group (Fig 1). This association was not consistent after adjustment for confounders (S2 Table). Expecting fathers with a history of previous depression more often reported anxiety (21.8% vs. 2.7%, OR = 10.2, CI = 9.5–10.9, p < 0.001) or depression (13.7% vs. 1.3%, OR = 12.6, CI = 11.5–13.8, p < 0.001) during pregnancy compared to those without previous depression, and with no significant difference between fathers with and without epilepsy. Anxiety/depression did not differ in FWE who reported expecting their first child vs. FWE with children from before.

**Fig 2:**
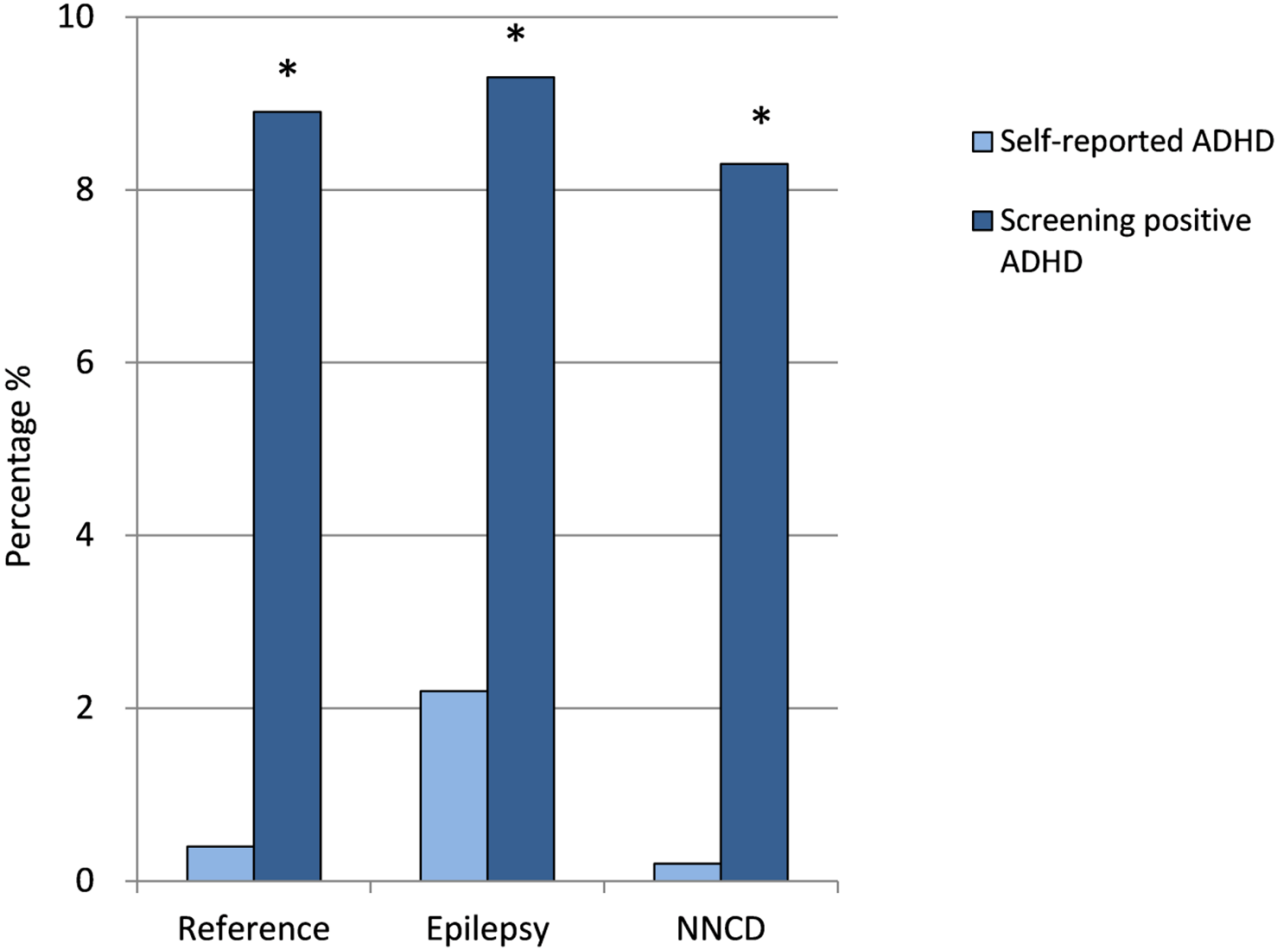
Self-reported diagnosis of ADHD vs. screening positive for ADHD symptoms in the epilepsy group, the reference group without epilepsy, and the group with non-neurological chronic disorders (NNCD). *Level of significance < 0.001.

ADHD was the second most common screening-positive diagnosis after previous depression among all the expecting fathers (Fig 1 and S2 Table). No difference in prevalence was found between men with and without epilepsy, or between FWE and fathers with NNCD. Screening-positive ADHD showed a higher prevalence than self-reported ADHD in men both with and without epilepsy (Fig 2). For FWE 2.2% reported ADHD while 9.5% had a positive symptom score for ADHD (p < 0.001).

**Fig 3:**
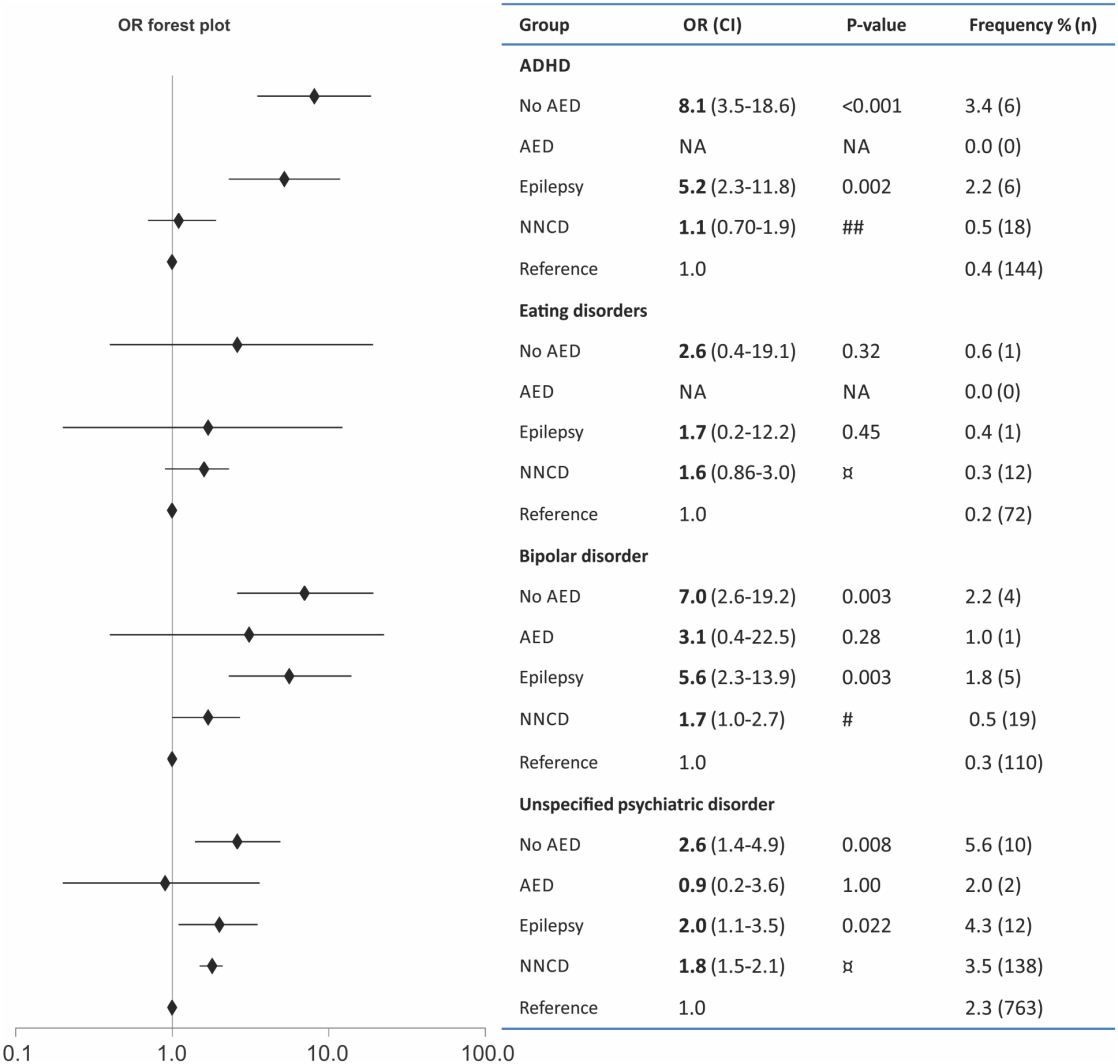
Frequencies for self-reported diagnoses of ADHD, eating disorders, bipolar disorder and other (unspecified) psychiatric disorders in fathers with epilepsy with and without use of antiepileptic drugs (AEDs) compared to a reference group without epilepsy. Unadjusted p-values and odds ratio (OR) are given for these comparisons. Fathers with non-neurological chronic disorders (NNCD) served as an additional internal control group. Significant difference between the NNCD versus ‘Epilepsy all’ groups: #p < 0.05; ##p < 0.01. ¤ No significant difference between the NNCD versus ‘Epilepsy all’ groups. CI, confidence interval; NA, not applicable.

A self-reported diagnosis of psychiatric disease was more frequent among FWE compared to the reference group (6.9% vs. 3.1%, p < 0.001), but not compared to those with NNCD (6.9% vs. 4.5%, p = 0.076). Psychiatric diagnoses were most common in AED-untreated epilepsy (Fig 3), 9.0% of AED-untreated (p < 0.001) and 3.0% of AED treated men (p = 1.00). ADHD was the most common self-reported psychiatric disorder, and both ADHD and bipolar disorder were increased in FWE compared to both reference groups (Fig 3). After adjustment for confounders, the OR of reporting ADHD was 3.2 in all FWE (p = 0.014) and 5.1 in the untreated group (p = 0.001), and OR for bipolar disorder was 4.1 in all FWE (p = 0.007) and 4.9 in the untreated group (p = 0.008) compared to the references (complete list of adjusted frequency for diagnoses in S3 Table). Other, unspecified psychiatric disorders were also more common among FWE compared to the references. In the untreated FWE we found OR = 2.6 (p = 0.004) after adjustment for confounders. No difference was found between FWE and the NNCD group.

### Self-esteem and satisfaction with life

Low self-esteem and low satisfaction with life were more common among FWE (Table 2). After adjustment for confounders, the association for low satisfaction with life remained significant (Table 2).

**Table 2:** Percentage and number (n) of individuals with low self-esteem and low satisfaction with life among fathers with epilepsy with and without use of antiepileptic drugs (AEDs) compared to a reference group without epilepsy. Fathers with non-neurological chronic disorders (NNCD) served as an additional internal control group.

		Unadjusted		Adjusted	
Group	% (n)	p-Value	OR (CI)	p-Value	OR (CI)
**Low Self Esteem**					
References	1.3 (980)				
NNCD	1.8 (145)	¤	-	-	-
Epilepsy	2.5 (16)	0.011	**1.9** (1.2–3.1)	0.083	**1.6** (0.94–2.6)
AED	2.5 (6)	0.14	**1.9** (0.85–4.3)	0.47	**1.4** (0.59–3.1)
No AED	2.5 (10)	0.045	**1.9** (1.0–3.5)	0.093	**1.7** (0.91–3.3.)
**Low Satisfaction With Life**					
References	0.7 (549)				
NNCD	1.0 (84)	¤	-	-	-
Epilepsy	1.7 (11)	0.010	**2.3** (1.3–4.3)	0.021	**2.1** (1.1–3.8)
AED	2.5 (6)	0.009	**3.5** (1.5–7.9)	0.018	**2.7** (1.2–6.2)
No AED	1.2 (5)	0.23	**1.6** (0.69–4.1)	0.31	**1.6** (0.65–3.9)

^¤^ No significant difference between the NNCD versus ‘Epilepsy all’ groups.

OR, unadjusted odds ratio; CI, confidence interval.

### Social characteristics and adverse life events

FWE reported a higher frequency of adverse social characteristics compared to the non-epilepsy reference group (Table 1). Low income, unemployment due to disability, and financial problems were more common among FWE compared to the references without epilepsy, and to NNCD (Table 1). The proportion of FWE reporting sick leave for more than 8 weeks yearly was higher than in men without epilepsy, but not compared to NNCD.

Adverse life events such as serious illness, having experienced physical violence, financial problems and conflict with family/friends/neighbors during the last 12 months, was more common among FWE compared to the reference group without epilepsy and the NNCD group (Table 3).

**Table 3:** Percentage and number (n) of individuals with various adverse life events in fathers with epilepsy with and without use of antiepileptic drugs (AEDs) compared to a reference group without epilepsy. Fathers with non-neurological chronic disorders (NNCD) served as an additional internal control group.

	References (n = 33944) ^1^	NNCD (n = 3959, 11.7%)^1^	Epilepsy all (n = 277, 0.8%)^1^			Epilepsy with AED (n = 99, 0.3%)^1^			Epilepsy without AED (n = 178, 0.5%)^1^		
Life events^1^	% (n)	% (n)	% (n)	p-Value	OR (CI)	% (n)	p-Value	OR (CI)	% (n)	p-Value	OR (CI)
Problems at work	27.7 (9222)	31.3 (1218)	30.0 (82)	0.39	**1.1** (0.86–1.5)	33.0(32)	0.25	**1.3** (0.84–2.0)	28.4 (50)	0.84	**1.0** (0.75–1.4)
Financial problems	15.1 (5040)	19.1 (746)^#^	25.2 (69)	<0.001	**1.9** (1.4–2.5)	26.8 (26)	0.001	**2.1** (1.3–3.2)	24.3 (43)	0.001	**1.8** (1.3–2.5)
Divorce/separation	2.1 (712)	2.5 (99)	2.6 (7)	0.64	**1.2** (0.57–2.6)	0.00 (0)	NA	NA	4.0 (7)	0.11	**1.9** (0.88–1.0)
Personal conflicts	16.7 (5563)	19.6 (762)^#^	24.9 (68)	<0.001	**1.7** (1.3–2.2)	29.9 (29)	0.001	**2.1** (1.4–3.3)	22.2 (39)	0.053	**1.4** (0.99–2.0)
Concerns about baby	10.3 (3433)	11.5 (450)	12.8 (35)	0.19	**1.3** (1.0–1.8)	13.4 (13)	.032	**1.3** (0.75–2.4)	12.5 (22)	0.34	**1.2** (0.79–1.9)
Serious injury/illness	4.3 (1438)	8.2 (321)	11.0 (30)	<0.001	**2.7** (1.8–3.9)	18.6 (18)	<0.001	**5.0** (3.0–8.4)	6.9 (12)	0.10	**1.6** (0.91–2.9)
Close relative injured/ill	17.8 (5936)	21.4 (834)	19.3 (53)	0.52	**1.1** (0.82–1.5)	15.5 (15)	0.53	**0.84** (0.49–1.5)	21.5 (38)	0.21	**1.3** (0.88–1.8)
Traffic accident/fire/ robbery	1.8 (613)	2.3 (91)	2.6 (7)	0.38	**1.4** (0.66–3.0)	3.1 (3)	0.27	**1.7** (0.54–5.4)	2.3 (4)	0.57	**1.2** (0.46–3.3)
Lost someone close	11.5 (3828)	12.7 (497)	14.3 (39)	0.15	**1.3** (0.91–1.8)	11.3 (119	0.96	**1.0** (0.53–1.8)	15.9 (28)	0.067	**1.5** (0.97–2.2)
Forced to sexual activity	0.2 (80)	0.4 (16)	0.00 (0)	NA	NA	0.00 (0)	NA	NA	0.00 (0)	NA	NA
Exposed to physical violence	1.5 (488)	2.0 (77)	3.6 (10)	0.008	**2.5** (1.3–4.8)	1.0 (1)	1.000	**0.70** (0.097–5.0)	5.1 (9)	0.001	**3.6** (1.8–7.1)

Significant difference between the NNCD versus ‘Epilepsy all’ groups:

^#^p < 0.05.

OR, unadjusted odds ratio; CI, confidence interval; SD, standard deviation; NA, not applicable.

^1^. Data only from questionnaire version D and E.

### Polytherapy and monotherapy

Complete crude frequencies with unadjusted OR and p-values for diagnoses and screening tools in the polytherapy and three monotherapy groups are found in S4 Table. Most observations were too rare to give significant numbers. In polytherapy-treated FWE there was one observation of bipolar disorder (7.7%, OR = 25.4, p = 0.042). In the VPA group six fathers reported anxiety (10.3%, OR = 2.4, p = 0.049) and three reported low satisfaction with life (5.2%, OR = 7.4, p = 0.009).

## Discussion

### Key findings

FWE more often had symptoms of anxiety and depression during pregnancy compared to both men without epilepsy and to men with other chronic disorders. The risk-estimates of psychiatric symptoms were similar for AED treated and untreated epilepsy groups. FWE also had an increased risk of ADHD, bipolar disorder, and other psychiatric disorders compared to both reference groups. Low self-esteem and low satisfaction with life was associated with epilepsy, as were adverse social aspects and life events.

### Strengths and limitations

This is the first study to compare mental health and socioeconomic conditions between expecting fathers with and without epilepsy. The cohort was collected from the general population and included both AED-treated and untreated epilepsy, reducing the risk of selection bias commonly associated with institutional-based cohorts. The reference group represents a large and heterogeneous population, leading to risk estimates that are more clinically applicable than in studies including only healthy controls. The data on socioeconomic aspects, and the broad spectrum of disorders recorded in MoBa, provides the possibility to adjust for confounders, and to compare epilepsy with other chronic disorders. Only 36.9% of the expecting fathers with epilepsy reported being treated with AED. This illustrates that most of them do not have active epilepsy with high risk of new seizures, but rather a history of previous epilepsy. This history is regarded as so relevant for their present health that they report it as a diagnosis. The fraction of individuals with a history of epilepsy using AED at present is in line with reports from other population-based registry studies [23, 24], which also demonstrated that AED treatment increases with increasing age. The mean age in our study population was 32 years. A near 100% validity for both the epilepsy diagnosis and use of medication has been shown for women with AED treated epilepsy in MoBa. Patients not treated with AEDs generally had inactive epilepsy [25]. Also, the prevalence of epilepsy is within the expected range for Western countries for both women and men in MoBa [26, 27]. The MoBa participation rate of 40.6% is as expected for large-scale population based cohorts [28]. Systematic bias caused by non-participants could be a concern, and a validation study on women in MoBa showed that they were slightly biased towards more favorable socioeconomic factors [29]. However, the same study found that exposure-outcome-rates were not affected by non-respondents or self-selection. Thus, comparisons between FWE and the reference groups are considered to be valid. The reported psychiatric diagnoses have not been validated, but complete versions of all screening tools applied have been validated.

A limitation of the present study is the lack of follow-up of the fathers post-partum. Post-partum health information would have been valuable to assess the specific effect of pregnancy. Information on type of epilepsy and seizure frequency would have determined epilepsy-specific effects in more detail.

### Interpretations

Depression and anxiety were more common in FWE compared to fathers without epilepsy. New onset of such symptoms during partner’s pregnancy was also more common in FWE, indicating that pregnancy constitutes a particular risk for mental health in epilepsy. Depression is the psychiatric disorder most commonly associated with epilepsy [30], and the association between epilepsy and anxiety is stronger than for other chronic disorders [31]. Newer studies suggest that expecting fathers are at risk of experiencing peri-partum depression and anxiety in relation to birth and life changes [2–4]. In fathers with epilepsy this may add to the burden of living with a chronic disorder. During childhood, restrictions and overprotection can cause stigma [8, 32]. Social insecurity and anxiety can affect friendships and social networks, the chance to find a life partner, choice of education, and work possibilities. Low income and lack of financial security were common in FWE, and both factors were associated with depression and anxiety. Private economic budgets become tighter with a new child, and this could worsen symptoms of anxiety and depression [33, 34]. Unemployment and long term sick leave, also more common in FWE, may lead to loss of social connections and isolation, causing depression, and anxiety. Psychic and emotional distress may decrease the probability of returning to work, causing a vicious circle. We found that previous depression was an indicator of both depression and anxiety during pregnancy. Former studies have found prenatal paternal depression to be predictive of postnatal depression [35, 36], and postnatal depression correlated with emotional and behavioral problems in the child [37]. As father’s mental health is a protective factor against depression in both mother and child [5], detecting symptoms of mental distress in the father early in pregnancy is important. Use of screening tools for symptoms of depression and anxiety represents an easy intervention.

We found a rather moderate prevalence of psychiatric disorders compared to former studies on non-pregnant persons with epilepsy [30, 38]. A plausible explanation is that pregnancy represents a stabilizing event, and that men with a pregnant partner represent a selection towards better health with less mental complaints. A partner may be both a test and a proof of qualities such as social skills and accepted behavior, and provides support for a chronic health condition [39]. The prevalence of psychiatric disorders reported in the present study may also reflect modern treatment and follow-up. Another explanation could theoretically be that psychiatric symptoms are interpreted as a consequence of the epilepsy manifestation, or as side-effects from AEDs [40], and therefore not reported as separate symptoms or diagnoses. Still, epilepsy in expecting fathers was associated with more adverse life events, psychiatric symptoms and self-reported psychiatric diagnoses. These results were in accordance with two previous surveys on pregnant women with epilepsy in MoBa [12, 27], where the women reported higher levels of depression, anxiety and adverse social circumstances. Although pregnancy is considered a positive experience, it can also trigger stress, anxiety and depression [35, 41], adding to an already increased vulnerability of psychiatric comorbidity in epilepsy [7].

Our study revealed an increase of self-reported ADHD in expecting FWE compared to the reference groups, and more specifically in the AED-untreated group. ADHD is reported with increased frequency in children with epilepsy [42], and a recent study claimed that ADHD symptoms occurred in nearly 20% of adult epilepsy [43]. The lower prevalence in our study probably reflects a more accurate prevalence in a population of otherwise healthy young men recruited from the general population. Furthermore, men with more severe psychiatric challenges may be less likely to have children. However, the rate of screening-positive ADHD symptoms was higher than the self-reported diagnose. The discrepancy between the rate of self-reported diagnosis and symptoms could indicate that ADHD is underestimated or undiagnosed. Alternatively, ASRS as a screening instrument may be too sensitive.

The correlation between bipolar disorder and epilepsy remained significant after logistic regression, indicating an independent association, supported by previous reports on increased frequency of bipolar disorder in epilepsy [44].

The risk of low self-esteem and low satisfaction with life were twice as high in FWE compared to the references without epilepsy, but not compared to NNCD. Depression, anxiety and perceived stigma are important predictors of quality of life and self-esteem [34]. Having a partner is, however, important for quality of life, feeling of security and self-confidence, and this could explain the overall high satisfaction among this group with a pregnant partner.

Low income, unemployment and long term sick leave were unfavorable aspects associated with epilepsy. Adverse life events, such as serious illness, physical violence, financial problems and conflicts with other people were also related to epilepsy and more common than in NNCD. Life style aspects such as smoking and drinking did not differ between men with and without epilepsy. Health authorities recommend avoiding tobacco and alcohol during pregnancy, and pregnant women tend to moderate their smoking, drinking and nutrition habits. This may influence their partner in a positive way, accounting for the similarities between the groups with and without epilepsy, this differing from former studies on epilepsy outside pregnancy [9].

Self-reported psychiatric disorders were more common in AED-untreated FWE. This is in line with our previous study on women in MoBa [27]. As AED treatment could indicate more severe epilepsy, our finding was interesting. Several AEDs are used in the treatment of psychiatric disorders [45]. Such drugs could have a modulating effect on psychiatric comorbidity in epilepsy.

## Conclusions

Men with epilepsy are at higher risk of depression, anxiety and other psychiatric disorders in relation to partner’s pregnancy compared to men without epilepsy. They have an increased risk also for ADHD and bipolar disorder compared to men with other chronic disorders. Adverse socioeconomic status, low self-esteem, and low satisfaction with life are also more common. We suggest that expecting fathers with epilepsy should be given more attention early in pregnancy, in particular regarding symptoms of depression and anxiety. The follow-up and treatment of psychiatric comorbidity is not only relevant to the expecting fathers, but also to their family and babies. The use of screening tools to assess comorbidity in epilepsy may be particularly useful to identify those at risk, as psychiatric diagnoses appear to be underreported.

## Supporting Information

S1 Table Variables used for Life Time Major Depression (LTMD), short version of Hopkin’s Symptom Checklist (HSCL), Adult ADHD Self Report Scale (ASRS), Rosenberg’s Self-Esteem Scale (RSES), and Satisfaction With Life Scale (SWLS). 1. Response option 1–4: “Not bothered”, “A little bothered”, “Quite bothered”, “Very bothered”. 2. Response option 1–5: “Never”, “Seldom”, “Sometimes”, “Often”, “Very often”. 3. Response option 1–4: “Strongly agree”, “Agree”, “Disagree”, “Strongly disagree”. 4. Response option 1–7: “Disagree completely”, “Disagree”, “Disagree somewhat”, “Neither nor”, “Agree somewhat”, “Agree”, “Agree completely”.
(DOCX)

S2 Table Frequencies for symptoms of ADHD tested with ASRS, previous depression tested with LTMD, current depression tested with SCL_D and current anxiety tested with SCL_A in fathers with epilepsy with and without use of antiepileptic drugs (AEDs) compared to a reference group without epilepsy. Unadjusted and adjusted p-values and odds ratios (OR) are given for these comparisons. Fathers with non-neurological chronic disorders (NNCD) served as an additional internal control group. ¤ No significant difference between the NNCD versus ‘Epilepsy all’ groups. CI, confidence interval; SD, standard deviation; ASRS, Adult ADHD Self Report Scale; LTMD, Lifetime Major Depression Scale; SCL_D, Hopkins Symptom Check List for current depressive symptoms; SCL_A, Hopkins Symptom Check List for current anxiety symptoms.
(DOCX)

S3 Table Frequencies for self-reported diagnoses of ADHD, eating disorders, bipolar disorder and other (unspecified) psychiatric disorders in fathers with epilepsy with and without use of antiepileptic drugs (AEDs) compared to a reference group without epilepsy. Unadjusted and adjusted p-values and odds ratio (OR) are given for these comparisons. Fathers with non-neurological chronic disorders (NNCD) served as an additional internal control group. Significant difference between the NNCD versus ‘Epilepsy all’ groups: #p < 0.05; ##p < 0.01. ¤ No significant difference between the NNCD versus ‘Epilepsy all’ groups. CI, confidence interval; NA, not applicable.
(DOCX)

S4 Table Frequency of psychiatric symptoms (ADHD tested with ASRS, previous depression tested with LTMD, current depression tested with SCL_D and current anxiety tested with SCL_A), psychiatric diagnoses (ADHD, eating disorder, bipolar, unspecified psychiatric disorders), low satisfaction with life (SWLS) and low self-esteem (RSES) in fathers with epilepsy treated with antiepileptic drug (AED) polytherapy or monotherapy, compared to a reference group without epilepsy. Unadjusted p-values and odds ratios (OR) are given for these comparisons. CI, confidence interval; SD, standard deviation; NA, not applicable; ASRS, Adult ADHD Self Report Scale; LTMD, Lifetime Major Depression Scale; SCL_D, Hopkins Symptom Check List for current depressive symptoms; SCL_A, Hopkins Symptom Check List for current anxiety symptoms; SWLS, Satisfaction With Life Scale” defined as score ≤ 9; RSES, Rosenberg Self-esteem scale.
(DOCX)
