# Supplementary material for: Psychiatric Comorbidity, Social Aspects and Quality of Life in a Population-Based Cohort of Expecting Fathers with Epilepsy
Source: PLoS One. 2015 Dec 4;10(12):e0144159. doi: 10.1371/journal.pone.0144159 (PMC4670115; doi:10.1371/journal.pone.0144159)
Supplement: S1 Table — 1. Response option 1–4: “Not bothered”, “A little bothered”, “Quite bothered”, “Very bothered”. 2. Response option 1–5: “Never”, “Seldom”, “Sometimes”, “Often”, “Very often”. 3. Response option 1–4: “Strongly agree”, “Agree”, “Disagree”, “Strongly disagree”. 4. Response option 1–7: “Disagree completely”, “Disagree”, “Disagree somewhat”, “Neither nor”, “Agree somewhat”, “Agree”, “Agree completely”. (DOCX) [file pone.0144159.s001.docx]

**S1 Table**. Variables used for Life Time Major Depression (LTMD), short version of Hopkin’s Symptom Checklist (HSCL), Adult ADHD Self Report Scale (ASRS), Rosenberg´s Self-Esteem Scale (RSES), and Satisfaction With Life Scale (SWLS).

| **Variables** | **Response options** |  |
| --- | --- | --- |
| **Life Time Major Depression** |  |  |
| *Have you ever experienced the following for a period of 2 weeks or more?:* | |  |
| Felt depressed, sad | No/ Yes |  |
| Had problems with appetite or eaten too much | No/ Yes |  |
| Been bothered by lack of energy | No/ Yes |  |
| Blamed yourself and felt worthless | No/ Yes |  |
| Had problems with concentration or had problems making decisions | No/ Yes |  |
| Had at least 3 of the problems named above simultaneously | No/ Yes |  |
| Was there a particular reason for this? | No/ Yes |  |
| **Hopkins Symptom Checklist** |  |  |
| *Have you been bothered by any of the following during the past two weeks?:* |  |  |
| Feeling fearful | 4 (1-4)^1^ | |
| Nervousness or shakiness inside | 4 (1-4) ^1^ | |
| Feeling hopeless about the future | 4 (1-4)^1^ | |
| Feeling blue | 4 (1-4)^1^ | |
| Worrying too much about things | 4 (1-4)^1^ | |
| Feeling everything is an effort | 4 (1-4)^1^ | |
| Feeling tense or keyed up | 4 (1-4)^1^ | |
| Suddenly scared for no reason | 4 (1-4)^1^ | |
| **Adult ADHD Self Report Scale** |  | |
| *Feeling of anxiety and restlessness in the last six months:* | | |
| How often do you have problems completing the final aspects of a task when the challenging part is already done? | 5 (1-5)^2^ | |
| How often do you have problems putting things in the right order when you are involved in tasks that require organization? | 5 (1-5)^2^ | |
| When you have a task which requires a great deal of careful preparation, how often do you avoid or put off starting it? | 5 (1-5)^2^ | |
| How often do you have problems remembering appointments or duties? | 5 (1-5)^2^ | |
| When you have to sit still for a long time, how often do you move your hands and feet in an agitated and restless way? | 5 (1-5)^2^ | |
| How often do you feel hyperactive and obliged to do things, as if you are being driven by an machine? | 5 (1-5)^2^ | |
| **Rosenberg´s Self-Esteem Scale** |  | |
| *What kind of perception do you have of yourself?:* | | |
| I have a positive attitude towards myself | 4 (1-4)^3^ | |
| I feel really useless at times | 4 (1-4)^3^ | |
| I feel that I don’t have much to be proud of | 4 (1-4)^3^ | |
| I feel that I’m a valuable person, on an equal footing with anyone else, at any rate | 4 (1-4)^3^ | |
| **Satisfaction With Life Scale** |  | |
| *Do you agree or disagree with the following statements?:* | | |
| My life is largely what I wanted it to be | 7 (1-7)^4^ | |
| My life is very good | 7 (1-7)^4^ | |
| I am satisfied with my life | 7 (1-7)^4^ | |
| To date, I have achieved what is important for me in my life | 7 (1-7)^4^ | |
| If I could start all over, there is very little I would do differently | 7 (1-7)^4^ | |
| **Life events** |  | |
| *Have you experienced any of the following during the last 12 months?* |  | |
| Problems at work |  | |
| Financial problems | No/ Yes | |
| Divorce/separation | No/ Yes | |
| Conflicts with family/friends/neighbours | No/ Yes | |
| Concerns about the baby | No/ Yes | |
| Serious personal injury/illness | No/ Yes | |
| Close relative being injured/ill | No/ Yes | |
| Involved in traffic accident/fire/robbery | No/ Yes | |
| Lost someone close | No/ Yes | |
| Forced into sexual activity, | No/ Yes | |
| Exposed to physical violence. | No/ Yes | |

1. Response option 1-4: “Not bothered”, “A little bothered”, “Quite bothered”, “Very bothered”.

2. Response option 1-5: “Never”, “Seldom”, “Sometimes”, “Often”, “Very often”.

3. Response option 1-4: “Strongly agree”, “Agree”, “Disagree”, “Strongly disagree”.

4. Response option 1-7: “Disagree completely”, “Disagree”, “Disagree somewhat”, “Neither nor”, “Agree somewhat”, “Agree”, “Agree completely”.
