# Supplementary material for: Psychiatric Comorbidity, Social Aspects and Quality of Life in a Population-Based Cohort of Expecting Fathers with Epilepsy
Source: PLoS One. 2015 Dec 4;10(12):e0144159. doi: 10.1371/journal.pone.0144159 (PMC4670115; doi:10.1371/journal.pone.0144159)
Supplement: S2 Table — ¤ No significant difference between the NNCD versus ‘Epilepsy all’ groups. CI, confidence interval; SD, standard deviation; ASRS, Adult ADHD Self Report Scale; LTMD, Lifetime Major Depression Scale; SCL_D, Hopkins Symptom Check List for current depressive symptoms; SCL_A, Hopkins Symptom Check List for current anxiety symptoms. (DOCX) [file pone.0144159.s002.docx]

**S2 Table.** Frequencies for symptoms of ADHD tested with ASRS, previous depression tested with LTMD, current depression tested with SCL_D and current anxiety tested with SCL_A in fathers with epilepsy with and without use of antiepileptic drugs (AEDs) compared to a reference group without epilepsy. Unadjusted and adjusted p-values and odds ratios (OR) are given for these comparisons. Fathers with non-neurological chronic disorders (NNCD) served as an additional internal control group.

|  | **Frequency** | **Unadjusted** |  | **Adjusted** |  |
| --- | --- | --- | --- | --- | --- |
| **Group** | **% (n)** | **p-Value** | **OR (CI)** | **p-Value** | **OR (CI)** |
| **ASRS (ADHD)** |  |  |  |  |  |
| No AED | 9.0 (16) | 0.94 | **1.0** (0.61-1.7) | 0.73 | **0.91** (0.53-1.6) |
| AED | 10.4 (10) | 0.60 | **1.2** (0.62-2.3) | 0.68 | **1.2** (0.59-2.2) |
| Epilepsy | 9.5 (26) | 0.71 | **1.1** (0.72-1.6) | 0.97 | **0.99** (0.65-1.5) |
| NNCD | 9.5 (370) | ¤ | **1.1** (1.0-1.2) | - | - |
| Reference | 8.9 (2948) |  | 1.0 |  |  |
| **LTMD (life time depression)** |  |  |  |  |  |
| No AED | 10.0 (41) | 0.96 | **0.99** (0.72-1.4) | 0.83 | **0.95** (0.58-1.5) |
| AED | 15.0 (36) | 0.012 | **1.6** (1.1-2.2) | 0.094 | **1.6** (0.92-2.7) |
| Epilepsy | 11.9 (77) | 0.14 | **1.2** (0.94-1.5) | 0.40 | **1.2** (0.8-1.7) |
| NNCD | 13.8 (1148) | ¤ | **1.5** (1.4-1.6) | - | - |
| Reference | 10.1 (7561) |  | 1.0 |  |  |
| **SCL_D (current depression)** |  |  |  |  |  |
| No AED | 3.7 (15) | 0.12 | **1.5** (0.90-2.5) | 0.35 | **1.4** (0.67-3.1) |
| AED | 4.2 (10) | 0.088 | **1.7** (0.92-3.3) | 0.20 | **1.8** (0.73-2.9) |
| Epilepsy | 3.9 (25) | 0.023 | **1.6** (1.1-2.4) | 0.13 | **1.6** (0.88-2.8) |
| NNCD | 3.5 (292) | ¤ | **1.5** (1.3-1.7) | - | - |
| Reference | 2.5 (1853) |  | 1.0 |  |  |
| **SCL_A (current anxiety)** |  |  |  |  |  |
| No AED | 7.1 (29) | 0.014 | **1.6** (1.1-2.3) | 0.057 | **1.7** (0.99-3.0) |
| AED | 6.7 (16) | 0.11 | **1.5** (0.91-2.5) | 0.16 | **1.7** (0.81-3.5) |
| Epilepsy | 7.0 (45) | 0.004 | **1.6** (1.2-2.1) | 0.018 | **1.7** (1.1-2.6) |
| NNCD | 6.2 (512) | ¤ | **1.4** (1.3-1.6) | - | - |
| Reference | 4.6 (3422) |  | 1.0 |  |  |

¤ No significant difference between the NNCD versus ‘Epilepsy all’ groups. CI, confidence interval; SD, standard deviation; ASRS, Adult ADHD Self Report Scale; LTMD, Lifetime Major Depression Scale; SCL_D, Hopkins Symptom Check List for current depressive symptoms; SCL_A, Hopkins Symptom Check List for current anxiety symptoms.
