# Supplementary material for: Psychiatric Comorbidity, Social Aspects and Quality of Life in a Population-Based Cohort of Expecting Fathers with Epilepsy
Source: PLoS One. 2015 Dec 4;10(12):e0144159. doi: 10.1371/journal.pone.0144159 (PMC4670115; doi:10.1371/journal.pone.0144159)
Supplement: S3 Table — Significant difference between the NNCD versus ‘Epilepsy all’ groups: #p < 0.05; ##p < 0.01. ¤ No significant difference between the NNCD versus ‘Epilepsy all’ groups. CI, confidence interval; NA, not applicable. (DOCX) [file pone.0144159.s003.docx]

**S3 Table.** Frequencies for self-reported diagnoses of ADHD, eating disorders, bipolar disorder and other (unspecified) psychiatric disorders in fathers with epilepsy with and without use of antiepileptic drugs (AEDs) compared to a reference group without epilepsy. Unadjusted and adjusted p-values and odds ratio (OR) are given for these comparisons. Fathers with non-neurological chronic disorders (NNCD) served as an additional internal control group*.*

|  | **Frequency** | **Unadjusted** |  | **Adjusted** |  |
| --- | --- | --- | --- | --- | --- |
| **Group** | **% (n)** | **p-Value** | **OR (CI)** | **p-Value** | **OR (CI)** |
| **ADHD** |  |  |  |  |  |
| No AED | 3.4 (6) | <0.001 | **8.1** (3.5-18.6) | 0.001 | **5.1** (2.0-13.1) |
| AED | 0.0 (0) | NA | NA | NA | NA |
| Epilepsy | 2.2 (6) | 0.002 | **5.2** (2.3-11.8) | 0.014 | **3.2** (1.3-8.0) |
| NNCD | 0.5 (18) | ## | **1.1** (0.70-1.9) | - | - |
| Reference | 0.4 (144) |  | 1.0 |  |  |
| **Eating disorders** |  |  |  |  |  |
| No AED | 0.6 (1) | 0.32 | **2.6** (0.4-19.1) | 0.35 | **2.6** (0.35-18.6) |
| AED | 0.0 (0) | NA | NA | NA | NA |
| Epilepsy | 0.4 (1) | 0.45 | **1.7** (0.2-12.2) | 0.63 | **1.6** (0.23-11.8) |
| NNCD | 0.3 (12) | ¤ | **1.6** (0.86-3.0) | - | - |
| Reference | 0.2 (72) |  | 1.0 |  |  |
| **Bipolar disorder** |  |  |  |  |  |
| No AED | 2.2 (4) | 0.003 | **7.0** (2.6-19.2) | 0.008 | **4.9** (1.5-15.7) |
| AED | 1.0 (1) | 0.28 | **3.1** (0.4-22.5) | 0.32 | **2.8** (0.38-20.2) |
| Epilepsy | 1.8 (5) | 0.003 | **5.6** (2.3-13.9) | 0.007 | **4.1** (1.5-11.2) |
| NNCD | 0.5 (19) | # | **1.7** (1.0-2.7) | - | - |
| Reference | 0.3 (110) |  | 1.0 |  |  |
| **Unspecified psychiatric disorders** |  |  |  |  |  |
| No AED | 5.6 (10) | 0.008 | **2.6** (1.4-4.9) | 0.004 | **2.6** ( 1.4-4.9) |
| AED | 2.0 (2) | 1.00 | **0.9** (0.2-3.6) | 0.85 | **0.88** (0.22-3.6) |
| Epilepsy | 4.3 (12) | 0.022 | **2.0** (1.1-3.5) | 0.025 | **2.0** (1.1-3.5) |
| NNCD | 3.5 (138) | ¤ | **1.8** (1.5-2.1) | - | - |
| Reference | 2.3 (763) |  | 1.0 |  |  |

Significant difference between the NNCD versus ‘Epilepsy all’ groups: #p < 0.05; ##p < 0.01.

¤ No significant difference between the NNCD versus ‘Epilepsy all’ groups. CI, confidence interval; NA, not applicable.
