# Supplementary material for: Psychiatric Comorbidity, Social Aspects and Quality of Life in a Population-Based Cohort of Expecting Fathers with Epilepsy
Source: PLoS One. 2015 Dec 4;10(12):e0144159. doi: 10.1371/journal.pone.0144159 (PMC4670115; doi:10.1371/journal.pone.0144159)
Supplement: S4 Table — CI, confidence interval; SD, standard deviation; NA, not applicable; ASRS, Adult ADHD Self Report Scale; LTMD, Lifetime Major Depression Scale; SCL_D, Hopkins Symptom Check List for current depressive symptoms; SCL_A, Hopkins Symptom Check List for current anxiety symptoms; SWLS, Satisfaction With Life Scale” defined as score ≤ 9; RSES, Rosenberg Self-esteem scale. (DOCX) [file pone.0144159.s004.docx]

**S4 Table.** Frequency of psychiatric symptoms (ADHD tested with ASRS, previous depression tested with LTMD, current depression tested with SCL_D and current anxiety tested with SCL_A), psychiatric diagnoses (ADHD, eating disorder, bipolar, unspecified psychiatric disorders), low satisfaction with life (SWLS) and low self-esteem (RSES) in fathers with epilepsy treated with antiepileptic drug (AED) polytherapy or monotherapy, compared to a reference group without epilepsy. Unadjusted p-values and odds ratios (OR) are given for these comparisons.

|  | **Reference** | **Polytherapy** | | | **Valproate monotherapy** | | | **Lamotrigine monotherapy** | | | **Carbamazepine monotherapy** | | |
| --- | --- | --- | --- | --- | --- | --- | --- | --- | --- | --- | --- | --- | --- |
|  | % (n) | % (n) | p | OR (CI) | % (n) | p | OR (CI) | % (n) | p | OR (CI) | % (n) | p | OR (CI) |
| **Psychiatric symptoms** |  |  |  |  |  |  |  |  |  |  |  |  |  |
| ASRS (ADHD) | 8.9 (2948 | 16.7 (2) | 0.29 | **2.1** (0.45-9.4) | 9.5 (2) | 0.71 | **1.1** (0.25-4.6) | 8.3 (2) | 1.00 | **0.93** (0.22-4.0) | 11.1 (3) | 0.73 | **1.3** (0.39-4.3) |
| Previous depression | 10.1 (7561) | 20 (6) | 0.12 | **2.2** (0.91-4.5) | 15.5 (9) | 0.17 | **1.6** (0.80-3.3) | 17.9 (7) | 0.11 | **1.9** (0.86-4.4) | 9.9 (9) | 0.95 | **0.98** (0.49-1.9) |
| Current depression | 2.5 (1853) | 3.4 (1) | 0.52 | **1.4** (0.19-10.3) | 5.2 (3) | 0.17 | **2.1** (0.67-6.9) | 5.1 (2) | 0.25 | **2.1**(0.51-8.8) | 2.2 (2) | 1.00 | **1.0** (0.22-3.6) |
| Current anxiety | 4.6 (3422) | 3.4 (1) | 1.00 | **0.75** (0.10-5.5) | 10.3 (6) | 0.049 | **2.4** (1.0-5.6) | 7.7 (3) | 0.43 | **1.7** (0.54-5.7) | 4.4 (4) | 1.00 | **0.97** (0.36-2.6) |
| **Psychiatric diagnoses** |  |  |  |  |  |  |  |  |  |  |  |  |  |
| ADHD | 0.4 (144) | 0 (0.0) | NA | NA | 0 (0.0) | NA | NA | 0 (0.0) | NA | NA | 0 (0.0) | NA | NA |
| Eating disorder | 0.2 (72) | 0 (0.0) | NA | NA | 0 (0.0) | NA | NA | 0 (0.0) | NA | NA | 0 (0.0) | NA | NA |
| Bipolar | 0.3 (110) | 7.7 (1) | 0.042 | **25** (3.2-197.29) | 0 (0.0) | NA | NA | 0 (0.0) | NA | NA | 0 (0.0) | NA | NA |
| Unspecified | 2.3 (763) | 0 (0.0) | NA | NA | 0 (0.0) | NA | NA | 4.2 (1) | 0.42 | **1.9** (0.25-13.9) | 4.5 (1) | 0.40 | **2.1** (0.28-15.3) |
| **Self-esteem and satisfaction with life** |  |  |  |  |  |  |  |  |  |  |  |  |  |
| Low self-esteem | 1.3 (980) | 3.3. (1) | 0.33 | **2.6** (0.35-19.0) | 1.7 (1) | 0.54 | **1.3** (0.18-9.4) | 5.1 (2) | 0.93 | **4.1** (0.98-16.9) | 1.1 (1) | 1.00 | **0.83** (0.12-6.0) |
| Low satisfaction  with life | 0.7 (549) | 3.4 (1) | 0.19 | **4.8** (0.66-35.6) | 5.2 (3) | 0.009 | **7.4** (2.3-23.6) | 0 (0.0) | NA | NA | 1.1 (1) | 0.49 | **1.5** (0.21-10.8) |

CI, confidence interval; SD, standard deviation; NA, not applicable; ASRS, Adult ADHD Self Report Scale; LTMD, Lifetime Major Depression Scale; SCL_D, Hopkins Symptom Check List for current depressive symptoms; SCL_A, Hopkins Symptom Check List for current anxiety symptoms; SWLS, Satisfaction With Life Scale” defined as score ≤ 9; RSES, Rosenberg Self-esteem scale.
